# Leveraging social capital: multilevel stigma, associated HIV vulnerabilities, and social resilience strategies among transgender women in Lima, Peru

**DOI:** 10.7448/IAS.20.1.21462

**Published:** 2017-02-27

**Authors:** Amaya G. Perez-Brumer, Sari L. Reisner, Sarah A. McLean, Alfonso Silva-Santisteban, Leyla Huerta, Kenneth H. Mayer, Jorge Sanchez, Jesse L. Clark, Matthew J. Mimiaga, Javier R. Lama

**Affiliations:** ^a^ Department of Sociomedical Sciences, Columbia University Mailman School of Public Health, New York, NY, USA; ^b^ The Fenway Institute, Fenway Health, Boston, MA, USA; ^c^ Division of General Pediatrics, Boston Children’s Hospital/Harvard Medical School, Boston, MA, USA; ^d^ Department of Epidemiology, Harvard T.H. Chan School of Public Health, Boston, MA, USA; ^e^ Universidad Peruana Cayetano Heredia, Lima, Peru; ^f^ Epicentro, Lima, Peru; ^g^ Asociación Civil Impacta Salud y Educación, Lima, Peru; ^h^ Department of Global Health, University of Washington, Seattle, WA, USA; ^i^ Centro de Investigaciones Tecnologicas, Biomedicas y Medioambientales, Lima, Peru; ^j^ Department of Medicine, Division of Infectious Diseases, University of California Los Angeles, Los Angeles, CA, USA; ^k^ The Institute for Community Health Promotion, Brown University School of Public Health, Providence, RI, USA

**Keywords:** Transgender women, social capital, HIV vulnerability, community strategies, Peru, resiliency

## Abstract

**Introduction**: In Peru, transgender women (TW) experience unique vulnerabilities for HIV infection due to factors that limit access to, and quality of, HIV prevention, treatment and care services. Yet, despite recent advances in understanding factors associated with HIV vulnerability among TW globally, limited scholarship has examined how Peruvian TW cope with this reality and how existing community-level resilience strategies are enacted despite pervasive social and economic exclusion facing the community. Addressing this need, our study applies the understanding of social capital as a social determinant of health and examines its relationship to HIV vulnerabilities to TW in Peru.

**Methods**: Using qualitative methodology to provide an in-depth portrait, we assessed (1) intersections between social marginalization, social capital and HIV vulnerabilities; and (2) community-level resilience strategies employed by TW to buffer against social marginalization and to link to needed HIV-related services in Peru. Between January and February 2015, 48 TW participated (mean age = 29, range = 18–44) in this study that included focus group discussions and demographic surveys. Analyses were guided by an immersion crystallization approach and all coding was conducted using Dedoose Version 6.1.18.

**Results**: Themes associated with HIV vulnerability included experiences of multilevel stigma and limited occupational opportunities that placed TW at risk for, and limited their engagement with, existing HIV services. Emergent resiliency-based strategies included peer-to-peer and intergenerational knowledge sharing, supportive clinical services (e.g. group-based clinic attendance) and emotional support through social cohesion (i.e. feeling part of a community).

**Conclusion**: This study highlights the importance of TW communities as support structures that create and deploy social resiliency-based strategies aimed at deterring and mitigating the impact of social vulnerabilities to discrimination, marginalization and HIV risk for individual TW in Peru. Public health strategies seeking to provide HIV prevention, treatment and care for this population will benefit from recognizing existing social capital within TW communities and incorporating its strengths within HIV prevention interventions. At the intersection of HIV vulnerabilities and collective agency, dimensions of bridging and bonding social capital emerged as resiliency strategies used by TW to access needed healthcare services in Peru. Fostering TW solidarity and peer support are key components to ensure acceptability and sustainability of HIV prevention and promotion efforts.

## Introduction

The Peruvian HIV epidemic is characterized by a disproportionate burden of infection among transgender women (TW), with HIV prevalence among TW ranging from 29.8% to 48.8% in Lima [1,2] compared to 0.4% among the general population [3]. Pervasive forms of social stigma and discrimination faced by TW in public and private spaces impact vulnerability to HIV acquisition and access to HIV prevention, treatment and care services. Notably, a number of key behavioural, social, structural and biological factors appear to drive these HIV disparities, including transactional sex practices [4], condomless receptive anal intercourse [4], low prevalence of HIV testing [1,5–7] and sexual contacts with unknown HIV serostatus partners [4]. Structural factors limiting TW access to healthcare are primarily due to the absence of gender identity legislation which restricts ability to change gender markers and legal name on official documents (i.e. *documento nacional de identidad)* [8,9]. Despite recent advances in understanding factors associated with HIV vulnerability among TW globally, limited scholarship examines how Peruvian TW cope with this reality and how existing community-level resilience strategies are enacted in the face of pervasive social and economic exclusion facing this community.

Globally, TW constitute a socially marginalized group, often with multiple intersecting stigmatized statuses beyond a gender identity that does not correspond with their sex assigned at birth (e.g. sex worker, economically disenfranchised, resource limited etc.) [1,10–12]. Key mechanisms reported in the literature to enact community-level resiliency strategies include strengthening of social connections and support mechanisms, peer education about needed medical information, community-based dissemination of specialized knowledge and other informal mechanisms for resource sharing [13–15]. Social connections, specifically within-group social cohesion (i.e. trust and reciprocal relationships), have been shown to buffer the adverse impact of mental health issues including depression and anxiety, both directly and indirectly related to coping strategies [13,16]. With regard to HIV prevention, lower levels of social support among TW have been associated with condomless anal sex [17], and peer-led models of HIV and STI prevention, testing and treatment for TW have been associated with increased engagement and retention in HIV care [18].

Social support is an important component of a larger group-level construct, social capital. While definitions of social capital vary and can be conceptually different, broadly, it describes the accumulation of actual and virtual resources that are derived from interactions and relationships between individuals nested in a community through social cohesion, support and within-group connectedness (i.e. solidarity) [19,20]. Scholarship supports the relationship between social capital and its health impacts [21–24]. Notably, social capital has been identified as a societal-level factor influencing HIV vulnerability [25–27]. Within a social determinant of health framework [28], social capital is described as a moderator with the ability to cross-cut macro, meso and interpersonal levels through which standard conceptualizations of social determinants (e.g. gender, socio-economic status, education, marital status etc.) operate [19]. Given the extreme social marginalization paired with the importance of trust and cooperative relationships among TW to access needed healthcare services and related knowledge [29–32], further understandings of *bonding social capital* (intra-group relations), *bridging social capital* (inter-group relations) and *linking social capital* (relations with institutions of power) are needed to inform public health strategies seeking to provide HIV prevention, treatment and care for this population.

This study builds on understandings of social capital as a social determinant of health and examines its relationship to HIV vulnerabilities among TW in Peru. Using qualitative methodology to provide an in-depth portrait, we assessed (1) intersections between social marginalization, multilevel stigma and HIV vulnerabilities and (2) community-level resilience strategies employed by TW to harness existing supports and link TW communities to needed HIV-related services in Peru.

## Methods

### Sampling and recruitment

The present analysis uses data from a formative study consisting of four focus group events (FG) conducted with TW (*N* = 48). Due to the expansive geographic area of Lima and to ensure broad geographic coverage in sampling, a purposive sampling approach [33] was implemented that stratified Lima by geographic region (i.e. *centro, cono norte, cono sur, cono este*) to maximize the variety of perspectives obtained. Recruitment of TW participants was conducted in collaboration with members of a Transgender Community Task Force (*n* = 7) who were convened based on past formative work. The Community Task Force was comprised of socially well-connected Peruvian TW community leaders who had prior experience engaging diverse local TW in Lima. Working in collaboration with study staff, meeting at least once a month, the Community Task Force was primarily responsible for recruitment and also ensured that all study procedures, instruments and protocols were culturally competent and gender affirming. To be eligible, potential participants needed to report that they were assigned a male sex at birth, self-identified as male-to-female TW or elsewhere on the trans-feminine continuum (e.g. “trans”, “transgender”, “travesti”) and were eighteen years or older. Participation was not restricted by reported HIV serostatus.

### Data collection

Conducted between January and February 2015, each FG lasted between 90 and 120 min and was comprised of the following components: written informed consent, a brief sociodemographic survey and a semi-structured FG discussion facilitated in Spanish. Discussion format covered three topic areas: existing HIV prevention, testing, care and treatment resources; perceived acceptability of integrating cross-sex hormone therapy administration into the HIV continuum of care and facilitators and challenges of healthcare delivery systems specific to TW needs. FG guides were pilot tested in one FG with TW (*n* = 13); pilot-testing data were not included in this report. FG events were conducted by a team of 2–3 research staff in the geographic region from which participants were recruited. All FG discussions were co-facilitated by two study staff members, including one cisgender female or male with extensive qualitative research experience and one transgender woman experienced in working with the TW community. Written informed consents were collected from each participant and, following consent, participants independently completed paper–pencil sociodemographic surveys. Participants received 20 Nuevos Soles (~US$ 8). Institutional Review Boards at Asociación Civil Impacta Salud y Educación in Lima, Peru and The Fenway Institute, Fenway Health, in Boston, MA approved the study.

### Analytic approach and data analysis

FG discussions were employed to obtain an understanding of community-level perspectives [34] about risk and vulnerabilities to HIV faced by Peruvian TW. FG probes specifically aimed to promote participant–participant interactions (e.g. incorporation of open-ended follow-up questions, facilitator briefly summarizing key points and allowing others in group to comment, incorporation of strategic pauses to prompt further discussion) and elicit an understanding of social discourses to detect collective and shared experiences. All FG were conducted in Spanish by native Spanish-speaking facilitators, audio recorded and transcribed verbatim. Guided by immersion crystallization, a qualitative approach which emphasizes both examining data and reflecting on the experience of being immersed in the data analysis, transcripts were analysed based on an inductive and deductive approach to identify themes and relationships between themes [35]. The analytic process was divided into multiple stages. A core group of 2–3 study staff, with previous experience in qualitative analyses, independently reviewed the transcripts, FG guides and sociodemographic surveys, and then coded each of the FG events. Initial sets of themes were independently assessed by two members of the research team who subsequently met to compare themes, discuss and reconcile any difference, and refine a set of codes and their definitions. A structured codebook was then developed transforming the themes into codes. The coding scheme was applied to an initial transcript by the two members of the team, and any discrepancies were discussed with a third member of the qualitative analysis group. All qualitative analysis was conducted using Dedoose Version 5.0.11 (Socio Cultural Research Consultants, LLC, www.dedoose.com).

## Results

Results are drawn from the narratives of 48 TW across four FG discussions. Half of participants were born in Lima, 8.3% in Piura, 8.3% in Iquitos and 6.3% in Pucallpa. Ages ranged from eighteen to forty-four years (mean = 29.1 years, SD = 7.5). Participants reported the following current living arrangements: living alone (27.1%), living with family (16.7%), living with a partner (31.3%) or living with one or more TW (14.6%). Almost half of the participants reported their sexual orientation as heterosexual (47.9%), gay (22.9%), bisexual (8.3%) and lesbian (2.1%); 8.3% did not provide a sexual orientation. Additionally, 10.4% of the participants reported trans/transwoman/*travesti* as their sexual orientation. Regarding means of income in the past three months, 60.4% (*n* = 29) reported engaging in sex work, 14.6% (*n* = 7) described formal or informal employment excluding sex work, 12.5% (*n* = 6) owned individual or group businesses (e.g. salon, restaurant, commerce etc.) and 4.2% stated that they received primary support from a spouse and/or partner.

**Figure 1.**
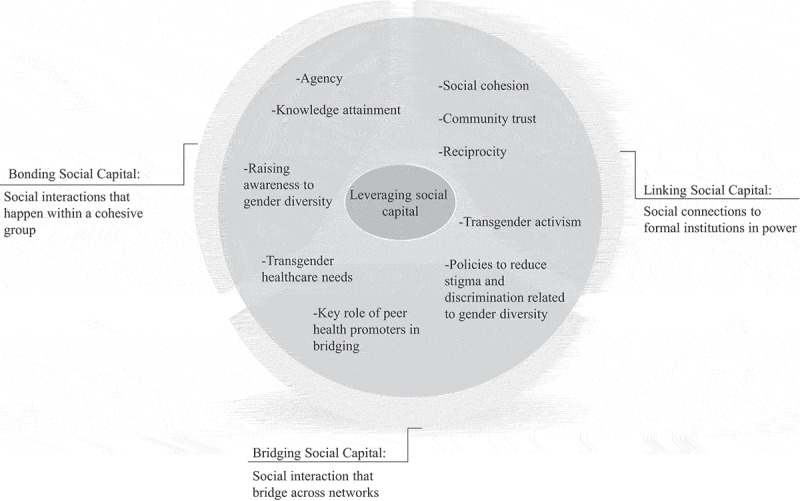
Visualization of existing social resilience-based strategies to circumnavigate barriers to HIV prevention and care leveraged by transgender women in Lima, Peru.

Results are presented in two thematic groupings: (1) intersections between social marginalization, multilevel stigma and HIV vulnerabilities (Table 1) and (2) community-level resilience strategies described by TW participants and how they are used to harness existing supports and to link TW communities to needed HIV-related services in Peru (Table 2). Additionally, analysis is presented to show how these emergent themes map onto the group-level construct of social capital, specifically characterized by *bonding social capital* (intra-group relations), *bridging social capital* (inter-group relations) and *linking social capital* (relations with institutions of power) (Figure 1).

**Table 1.** Illustrative quotes of intersecting multilevel stigma and associated HIV vulnerabilities among transgender women in Lima, Peru (*n* = 48)

No.	*Illustrative quotes*
1	“To a transgender woman, working on the street is not easy, it is not like we go there because we like it, it is the only way to survive” [FG #3]
2	“…there are many of us with who have studied but we are discriminated against, we are not accepted. What can I tell you, sometimes we drop out of school or work … because there is so much bullying and discrimination in businesses, institutes, schools, everywhere, that we quit and the only solution is to go to the street” [FG#4]
3	“…there is so much risk in the street, most of us that go to the street are not infected, the same clients know they have something wrong, but they do not want to use the condom so they offer you a considerable amount [to not use a condom” [FG #3]
4	“…depends on what you’ve gone through in your life, how you’ve lived it, if you’ve lived it badly, if you haven’t used some kind of protection. If you didn’t protect yourself, it’s in your conscience if you become infected” [FG#2]
5	“Most *travestis* go to [clinics to] receive condoms because this is [their] working material, but nowadays, the medical centers do not want to give condoms to sex workers” [FG#4]
6	“We worry and fear about the place where you are going to be attended … if I go to the doctor they will reprimand me, it happens, and that’s why sometimes we are afraid of going to the hospital” [FG#2]
7	“Fear you feel knowing that they [healthcare providers] are going to treat you badly just because you have that disease [HIV]” [FG#1]
8	“…they [medical providers and staff] make us feel uncomfortable” [FG#3]
9	“from the moment I get there I feel inhibited. I think that they are going to treat me badly and I don’t feel comfortable” [FG#1]
10	“ ‘You man, you man’ … calling you by the name recorded on your DNI [National Identity Card]” [FG#2]
11	“You go to the health center, people … see us as women, but they have the bad habit of calling us by our legal name instead of … our last name. Then, the girls feel bad…” [FG#3]

**Table 2.** Illustrative quotes of how social capital is utilized to enact social resiliency-based strategies  among transgender women in Lima, Peru (*n* = 48)

No.	*Illustrative quotes*
12	“We have learned to defend ourselves and to fight for respect together” [FG#3]
13	“I go with one friend and together we get tested” [FG#2]
14	“It would be nice if when you go [to a healthcare center] you know you will receive a nice or kind treatment…. [but] we go in groups of 3-5 because we know what type of treatment we are going to receive” [FG#1]
15	“…as a defense mechanism … because if there is bullying, there is always someone there. [You] feel less vulnerable” [FG# 3]
16	“When you go with the promoter the service is quicker … [and] without problems” [FG#4]
17	“…all of us have come with a promoter to healthcare centers. No one goes alone” [FG#2]
18	“Because you can tell them [promoters] your problems and they will guide you since they know more about HIV” [FG#1]
19	“They call me by my legal name and I stay seated, looking at the ceiling, until [the nurse] is bothered or I am bothered, so I look for her boss and tell him what is happening. But, with the [younger] girls’ case it is not like that. [The nurses] call her by their legal name and they automatically get up and leave … [but] when they are in group, it is different, and they [her friends] say: ‘Hey, what is wrong with you? She is my friend’” [FG#4]
20	“United we must fight for laws. For example, in Argentina, you can have a female name, and also, on your ID you can select your gender as female without surgery” [FG#1]
21	“…we have advantage … as activists … to learn issues of the law” [FG#4]
22	“As an activist, I like [younger] girls to join us because they are learning law issues, they are learning that they must not be stigmatized” [FG#3]

FG narratives describing vulnerabilities to HIV acquisition were frequently linked to dimensions of economic and social marginalization and expressed across multiple intersecting levels (Table 1). Notably, these contextual barriers are described as interwoven within societal structures and, also, within HIV prevention and care facilities. To this point, FG discussions illustrated the reality of engagement in sex work for many TW in Peru (Quote 1) and how this occupational backdrop is associated with HIV vulnerabilities (Quote 2). Pervasive social stigma and discrimination against TW were described as limiting opportunities for economic advancement irrelevant of other qualifications such as educational attainment (Quote 2). As such, the “choice” to engage in sex work is bounded by the availability of economic opportunities and the need to survive. In the Peruvian context, participants further discussed the associations between sex work and HIV vulnerabilities including power imbalances that influenced the ability to use HIV risk-reduction strategies, such as condom negotiation between sex worker and client (Quote 3).

Intersecting with the high prevalence of sex work, vulnerability to HIV infection included a discussion of multiple layers of marginalization and oppression (i.e. multilevel stigma) that limited TW engagement with existing HIV prevention, treatment and care services. For example, anti-sex-worker stigma (extending beyond TW who engage in sex work) and community-level transphobia (negative attitudes towards TW) were described as increasing HIV vulnerabilities (e.g. healthcare provider judgement, limited safe space to engage in street-based sex work, lack of legal protections for sex work). These themes were highlighted at an individual level not only exemplifying internalized stigma (Quote 4), but also within medical spaces (Quotes 5–11) and link to structural inequities in society broadly (Quote 10–11). For those TW that reported engaging in sex work, they discussed their occupation as a catalyst for routine contact with healthcare clinics, primarily to retrieve condoms (Quote 6). However, Quote 6 additionally shows the layered perceptions of sex-worker-based stigma and anti-transgender attitudes, in the form of policing who can receive condoms, that limit HIV prevention and care services within a medical space.

Narratives highlighted how, in this context, membership to or being labelled as transgender additionally represents a constellation of stigmatized statuses (e.g. sex worker, HIV positive etc.) regardless of whether or not the individual herself is living with HIV or engages in sex work. These overlapping stigmas were described as manifesting in anticipated fear about the quality of care and judgements they receive within a medical space when seeking HIV prevention and care (Quote 6–10). Specific to HIV stigma, beyond being labelled as HIV positive or experiencing HIV stigma, participants clearly provided an example of friends known to be living with HIV and how HIV stigma influenced the care they received (Quote 8). Jointly, these narratives highlight distinct, yet interrelated, dimensions of stigma faced due to being transgender.

Multilevel stigma rooted in anti-transgender attitudes (i.e. transphobia) was also described as taking many forms within a clinical space, including, but not limited to, misused pronouns (Quote 10) and behaviours to publically shame and/or flag discordance in gender makers among TW (i.e. calling patient by legal first name vs. preferred first name or last name alone) (Quote 11). While examples of transphobia were explicitly described at the meso-level in the healthcare setting, they link to structural-level barriers and policies. For example, these quotes refer to “legal name” and “national identity documents” showing broader structural inequities, namely the lack of legal protections for transgender people in Peru to change gender markers and names (that cross gender categories) on official documentation.

To cope with the reality of stigma, discrimination and vulnerability to HIV infection, both in daily lived realities and within healthcare settings, participants recounted several social resiliency-based strategies to leverage resources accessed through their networks (i.e. social capital) and to combat barriers described above. When assessed jointly, emergent social resiliency strategies were grounded in the group-level construct of social capital (Quotes 12–22) and, within this construct, also displayed unique dimensions of social capital: bonding, bridging and linking (see Figure 1).

In Figure 1, *bonding* social capital refers to the utilization of solidarity and social cohesion (i.e. a sense of belonging to a community, within community trust and reciprocity, willingness to support and share resources) to jointly overcome known barriers. For example, participants reported attending clinics in pairs or groups (Quote 12–15) as a buffer to protect against anticipated stigma in medical space or by staff. *Bridging* social capital refers to the use of key nodes to bridge and connect distinct networks. Peer navigators, while part of TW social networks, also manoeuver within biomedical spaces and thus are seen as a bridge to access needed HIV testing and care (Quotes 16–19). On a broader scale, *linking* social capital refers to the unified efforts of the collective (i.e. the broader TW community) to influence formal institutions of power. Participants described the use of activism to challenge broader forms of societal stigma and discrimination (Quotes 20–22).

Several of the social resilience-based strategies that emerged serve multiple functions to combat contextual barriers. The utilization of peer navigators, beyond bridging the heterogeneous networks (i.e. TW community members and healthcare staff), exemplifies the use of bonding social capital (i.e. social cohesion to promote knowledge and service attainment) [36]. Participants strongly endorsed community-leader peer navigation and described social cohesion as essential to navigating existing challenges when seeking HIV prevention and care (Quotes 16–17) or related information (Quote 18).

Descriptions of community leaders were not restricted to peer promoters. Multiple participants recounted that younger and/or less experienced TW who are new to the community often relied on the advice and guidance of older TW community leaders (Quote 19, 22). While descriptions of transgender leaders can be assessed as a form of bonding (e.g. strengthening within group cohesion) and bridging social capital (e.g. existing knowledge networks including and beyond biomedical services), they were also described as crucial to activism efforts and influencing formal institutes of power (i.e. linking social capital) specifically to advocate for a Peruvian gender identity law. Quotes highlighted the existence of cross-generational mentorship in guiding younger TW through increased awareness of transgender collectives and activism efforts across countries in South America (Quote 20).

## Discussion

This study highlights the importance of TW communities as support structures for individual TW in Peru through the creation of social resiliency-based strategies that deter and mitigate the impact of social vulnerabilities based on discrimination, marginalization and HIV risk. Narratives related to the social determinants of HIV vulnerability articulated by TW in this study parallel existing literature [13,37] and underscore the importance of pervasive social exclusion, fuelling economic marginalization, limiting employment to sex work for many and the collective association of these factors with HIV vulnerabilities [4].

In the context of multiple intersecting layers of social marginalization and oppression (i.e. multilevel stigma) that drive HIV infection in the community, narratives described common strategies of peer trust and cooperative relationships among TW that promote routine access to needed healthcare services, knowledge of HIV prevention and treatment strategies, and collaborative pathways to ameliorate shared HIV vulnerabilities. The strategies utilized by TW in Peru are best conceptualized using the group-level construct of social capital – the accumulation of resources derived from interactions and relationships between individuals nested in a community characterised by social cohesion, support and within-group connectedness (i.e. solidarity) [28]. Narratives underscore existing community-level expertise embedded within social networks that is actively being leveraged by a range of TW. Public health strategies seeking to provide HIV prevention, treatment and care for this population will benefit from incorporating this existing social capital within TW communities. However, greater attention needs to be paid to the various dimensions of social capital (i.e. bonding, bridging and linking) enacted by TW in Lima, Peru. Results complement emergent pedagogical frameworks such as prevention literacy [38] and empowering methodology [39] which seek to recognize and affirm community-level expertise by shifting the source of knowledge production from the public health worker/healthcare provider to TW who have already harnessed available tools and know what their HIV prevention needs are.

These findings should be interpreted with caution, in light of several limitations. We present data from a small qualitative study in Lima, Peru, and findings may not be generalizable. Participants in this study were recruited from traditionally impoverished areas of the city where access to basic resources, including clean water and electricity, is often limited potentially portraying one dimension of economic and social marginalization among TW in Lima. Social capital was not the primary focus of the study but emerged as a salient theme in qualitative analyses underscoring the centrality of this construct for TW. Additionally, these findings elucidate only positive dimensions of social capital among TW to mitigate HIV risk as these were the emergent themes from our data. Scholars seeking to further this line of research inquiry should also refer to the literature on HIV risk within social networks (i.e. pivotal role of “trans mothers” in initiating sex work) [15] to provide a more nuanced assessment of the relationship between social networks and HIV vulnerability among TW. Despite these limitations, our results provide an important first step in the Peruvian context and suggest the need for additional scholarship to understand and characterize the phenomena of social exclusion and social capital among TW, including the nature, scale and mechanisms of interactions between TW [40]. Such efforts will directly inform the development and refinement of public health interventions, practice, policy and social justice. Critical to this endeavour is the inclusion and integration of TW community perspectives to guide public health understandings and action [41].

## Conclusion

Inclusion and exclusion can be understood as a continuum characterized by unequal access to resources, capacities and rights which produce and sustain health disparities. Exclusion was a pervasive backdrop to the narratives presented here and should be conceptualized as a dynamic process driven by unequal power relationships that interact across different dimensions – economic, political, social and cultural – and on different levels, ranging from individual and household, to group, community and global [42]. Transphobia and stigma marginalize TW and push them “outside of the system”. Social exclusion therefore creates an imperative that TW communities find alternative ways to address their health needs and the unique social circumstances facing them.

The existing community-level response deployed by TW communities in Peru to combat social exclusion and marginalization (both of which are embedded and reproduced in Peruvian societal structures) leverages multidimensional social capital to create TW-specific social and cultural systems and community infrastructures. Figure 1 summarizes key themes which future scholars seeking to identify and target novel interventions for TW in Peru can build upon based on these findings. Resiliency-based strategies included: knowledge sharing not only between peers but also across generations (i.e. older TW transmit knowledge to younger TW); support at potential points of social tension and contact with discriminatory or marginalizing institutional structures (e.g. group-based clinic attendance); and emotional support that both draws on and further reinforces social cohesion (i.e. being part of a community). In conversation with literature reporting similar findings, the importance of generational knowledge transmission, for example, of a “trans mother” [15], illustrates an underdeveloped pathway which may be particularly important for public health interventions that seek to disseminate HIV prevention and health literacy within the existing social structures of TW. Adding to the central role of generational mentorship within Peruvian TW networks, these findings further illustrate the importance of within group mentorship to foster transgender collectives, solidarity and activism within Peru and suggest linkages to other networks in South America (i.e. Argentina).

At the intersection of HIV vulnerabilities and collective agency, social resilience emerges as a strategy used by TW to access needed healthcare services and a potential pathway to ameliorate HIV vulnerabilities in Peru. Public health attention is needed to recognise and further understand social capital as a social resiliency tactic deployed by TW to resist exclusionary practices and ensure a collective sense of belonging and community empowerment. Consistent with current literature [39,43], findings highlight that existing community-level expertise in tandem with fostering TW solidarity and peer support are potentially key to promoting the acceptability and sustainability of public health interventions across the HIV continuum of care for Peruvian TW.
